# Relationship between lipoprotein concentrations and short-term and 1-year mortality in intensive care unit septic patients: results from the HIGHSEPS study

**DOI:** 10.1186/s13613-021-00800-0

**Published:** 2021-01-19

**Authors:** Sébastien Tanaka, Jules Stern, Donia Bouzid, Tiphaine Robert, Monique Dehoux, Aurélie Snauwaert, Nathalie Zappella, Maxime Cournot, Brice Lortat-Jacob, Pascal Augustin, Enora Atchade, Alexy Tran-Dinh, Olivier Meilhac, Philippe Montravers

**Affiliations:** 1 Assistance Publique - Hôpitaux de Paris (AP-HP), Department of Anesthesiology and Critical Care Medicine, DMU PARABOL, Bichat-Claude Bernard Hospital, Paris, France; 2 Réunion Island University, French Institute of Health and Medical Research (INSERM), U1188 Diabetes Atherothrombosis Réunion Indian Ocean (DéTROI), CYROI Plateform, Saint-Denis de La Réunion, France; 3 Université de Paris, Paris, France; 4 Assistance Publique - Hôpitaux de Paris (AP-HP), Emergency Department, Bichat-Claude Bernard Hospital, Paris, France; 5 French Institute of Health and Medical Research (INSERM) U1137, Infection, Antimicrobials, Modelling, Evolution, Paris, France; 6 Assistance Publique - Hôpitaux de Paris (AP-HP), Biochemistry Department, Bichat-Claude Bernard Hospital, Paris, France; 7 French Institute of Health and Medical Research (INSERM) U1148, Laboratory for Vascular Translational Science, Paris, France; 8 Réunion Island University-Affiliated Hospital, Saint-Denis de la Réunion, France; 9 French Institute of Health and Medical Research (INSERM) U1152, Physiopathology and Epidemiology of Respiratory Diseases -ANR-10-LABX-17, Paris, France

**Keywords:** Sepsis, High-density lipoprotein, Low-density lipoprotein, Mortality, Intensive care unit, Outcome

## Abstract

**Background:**

High-density lipoproteins (HDLs), particles characterized by their reverse cholesterol transport function, display pleiotropic properties, including anti-inflammatory and antioxidant functions. Moreover, all lipoproteins (HDLs but also low-density lipoproteins (LDLs)) neutralize lipopolysaccharides, leading to increased bacterial clearance. These two lipoproteins decrease during sepsis, and an association between low lipoprotein levels and poor outcome was reported. The goals of this study were to characterize the lipid profile of septic patients hospitalized in our intensive care unit (ICU) and to determine the relationship with the outcome.

**Methods:**

A prospective observational study was conducted in a university hospital ICU. All consecutive patients admitted for septic shock or sepsis were included. Total cholesterol, high-density lipoprotein cholesterol (HDL-C), low-density lipoprotein cholesterol (LDL-C), and triglyceride levels were assessed at admission (day 1), at day 3, and at ICU discharge. When available, a prehospitalization lipid profile collected prior to the patient’s hospitalization was compiled. Short-term and 1-year prognostic outcomes were prospectively assessed.

**Results:**

A total of 205 patients were included. We found a decrease in HDL-C concentration between previous values and those at admission, followed by an additional decrease at day 3. At ICU discharge, the concentration was higher than that at day 3 but did not reach the concentration measured prior to hospitalization (prior HDL-C = 1.22 (1.04–1.57) mmol/l; day 1 HDL-C = 0.44 (0.29–0.70) mmol/l; day 3 HDL-C = 0.30 (0.25–0.48) mmol/l; and HDL-C at discharge = 0.65 (0.42–0.82) mmol/l). A similar trend was found for LDL-C (prior LDL-C = 2.7 (1.91–3.33) mmol/l; day 1 LDL-C = 1.0 (0.58–1.50) mmol/l; day 3 LDL-C = 1.04 (0.64–1.54) mmol/l; and LDL-C at discharge = 1.69 (1.26–2.21) mmol/l). Mixed models for repeated measures of lipoprotein concentrations showed a significant difference in HDL-C and LDL-C concentrations over time between survivors and nonsurvivors at day 28. An HDL-C concentration at admission of less than 0.4 mmol/l was associated with increased mortality at day 28 (log-rank test, *p* = 0.034) but not at 1 year (log-rank test, *p* = 0.24). An LDL-C concentration at admission of less than 0.72 mmol/l was associated with increased mortality at day 28 and at 1 year (log-rank test, *p* < 0.001 and *p* = 0.007, respectively). No link was found between prior lipid profile and mortality.

**Conclusions:**

We showed no relationship between the prehospitalization lipid profile and patient outcome, but low lipoprotein levels in the ICU were strongly associated with short-term mortality.

## Background

Sepsis remains an important cause of mortality in intensive care units (ICUs) despite recent better comprehension of its pathophysiology [1, 2]. Previous studies have suggested that during sepsis, multiple organ dysfunctions occur consecutive to major endothelial alterations, leading to microcirculation dysfunction, platelet and leukocyte activation, and coagulation pathway perturbations [3]. High-density lipoproteins (HDLs) represent a family of particles that are characterized by their ability to transport cholesterol from peripheral tissues back to the liver, which causes them to have a cardiovascular protective effect [4, 5]. These particles have some pleiotropic endothelioprotective properties, including anti-inflammatory, anti-apoptotic, and antioxidant functions [6–11]. Like all lipoproteins, another major property of HDLs is the capacity to neutralize lipopolysaccharides and increase their clearance [12–15]. Experimental studies testing both reconstituted HDL and apolipoprotein A-I mimetic peptide perfusion in animal models of septic shock demonstrated a protective effect of these HDL mimetics on mortality and a decrease in inflammatory parameters [16–20]. Clinical observations in numerous acute and chronic inflammatory pathologies, including sepsis, have shown a decreasing level of high-density lipoprotein cholesterol (HDL-C) [21–28]. Some studies found a negative correlation between HDL-C concentration and mortality [24–27], while other studies did not find a statistically significant relationship [23, 29–31].

Low-density lipoproteins (LDLs) are able to neutralize lipopolysaccharide [32, 33], and observational studies report that low-density lipoprotein cholesterol (LDL-C) levels can decrease by 30% in patients experiencing inflammatory states, such as sepsis [27, 34]. Walley et al. also demonstrated that low LDL-C is associated with poor prognosis during sepsis [35].

Objectives of the study were as follows:

- The first objective of the study was to establish the kinetics of lipid profiles over time (basal lipid assessment, admission level, 48 h, and day of discharge from the ICU).
- Second, the controversial results of the abovementioned studies regarding the link between mortality and lipoprotein concentrations led us to conduct a prospective observational study in our surgical ICU with the aim of characterizing the lipid profiles of septic patients and ultimately to seek a statistical link between lipoprotein concentrations and short-term and 1-year patient mortality.
- The last objective of this work was to determine whether there is a relationship between basal lipoprotein levels before hospitalization and ICU outcome.

## Methods

This was a prospective, observational monocentric study conducted in the surgical intensive care unit of Bichat Claude-Bernard University Hospital, Paris, France. Patients were recruited from May 2016 to April 2019. All patients admitted for septic shock or severe sepsis according to the criteria of the Surviving Sepsis Campaign were included [1]. Cirrhotic and immunocompromised patients (acquired immune deficiency syndrome or transplant surgery) were excluded from this study. This study was approved by the French Society of Anesthesiology and Critical Care Medicine Research Ethics Board (HIGHSEPS study, IRB 00010254).

Patient demographics, diagnosis, Simplified Acute Physiology Score II and Sepsis-related Organ Failure Assessment severity scores, and clinical data were collected prospectively. Regular medication use was also collected. In the case of habitual long-term statin use, i.e., starting before lipoprotein data collection, treatment was continued during the patient’s hospitalization. Renal function was assessed with the KDIGO AKI stage (Kidney Disease: Improving Global Outcomes Acute Kidney Injury stage) [36]. The percentage of patients receiving nutrition during the ICU stay was determined.

Data regarding the site of infection were also collected. ICU and in-hospital mortality at 28 days, 90 days, and 1 year, duration of mechanical ventilation, number of days living without mechanical ventilation at day 28, length of stay in the ICU and in the hospital, renal replacement therapy and vasopressor use, and SOFA score at admission, at 48 h, and on the day of discharge were collected. At admission (day 1), at 48 h (day 3), and on the day of discharge (discharge day), plasma concentrations of total cholesterol, HDL-C, LDL-C, and triglycerides were measured. These analyses were performed in the Biochemistry Laboratory of Bichat Claude-Bernard Hospital. Total cholesterol (TC), HDL-C, LDL-C, and triglyceride concentrations were determined by routine enzymatic assays (CHOL, HDL-C, and TRIG methods, Dimension VISTA® System, Siemens Healthineers™). The reference values for these assays were HDL-C > 1.40 mmol/l, total cholesterol 4.40 < N < 5.20 mmol/l, and triglycerides 0.50 < N < 1.7 mmol/l. According to the recommendations of the French National Authority for Health 2017 and the European Society of Cardiology 2016, LDL-C concentration targets have been established depending on vascular risk factors [37].

When a patient was included in this study, the physician in charge of the patient asked the patient himself or his family whether a complete lipid panel had been performed within the 2 years prior to the patient’s ICU admission. This assessment had to have been performed outside of any infectious episode. If available, these results were considered as the prehospitalization lipid test.

Concerning alimentation, intubated patients received early enteral feeding. When it was not possible, particularly in abdominal-onset sepsis, parenteral nutrition was administered temporarily. In nonintubated patients, oral feeding was given as early as possible.

### Statistical analysis

Categorical variables were compared using either the Chi-square test or Fisher’s exact test. Continuous variables were compared using Student's *t*-test (for parametric data) or the Mann–Whitney *U*-test (for nonparametric data), as appropriate. Repeated measures were compared using the Friedman test. Continuous variables were compared using Spearman's coefficient.

A mixed model for repeated measures of lipoprotein levels was built to predict mortality at day 28, with unstructured within-subject covariance. This model has the advantage of allowing unbalanced missing data and various within-subject covariance structures. Kaplan–Meier survival curves were constructed for the 28-day, 90-day, and 365-day periods after the onset of sepsis, and subgroups were compared with the log-rank test findings. Cut-offs of lipoproteins concentration used in the Kaplan–Meier were chosen from the optimal point on the ROC curves plotted to predict mortality at day 28 (data not shown).

Statistical analysis was performed using STATA version 15 (StataCorp LP, College Station, TX, USA) and GraphPad Prism 4.0 (GraphPad Software, La Jolla, CA, USA).

## Results

### a. Population

Two hundred five patients with sepsis were included prospectively and consecutively from May 2016 to April 2019. ICU mortality was 17%. Mortality at 28 days was also 17%. Mortality at 90 days and 1 year was 24% and 29%, respectively.

Table 1 shows the general characteristics of the patients, the etiology of sepsis, and the outcome of the patients.

**Table 1:** General characteristics of the population, outcome, and type of sepsis

	All patients (*n* = 205)	Patients dead at 28 days (*n* = 35)	Patients alive at 28 days (*n* = 170)	*p*
Male (n; %)	108; 52%	21; 60%	87; 51%	0.3595
Age (years), med (IQR)	63 (52–73)	67 (62–77)	63 (51–72)	0.0208
Weight (kg), med (IQR)	75 (64–90)	70 (60–86)	78 (65–90)	0.1593
Statin (*n*; %)	63; 31%	8; 22%	55; 32%	0.3183
Septic shock (*n*; %)	149; 73%	33; 22%	116; 78%	0.0008
Severe sepsis (*n*; %)	56; 27%	2; 4%	54; 96%	< 0.0001
Bacteremia (*n*; %)	53; 26%	12; 23%	41; 77%	0.1259
Intra-abdominal sepsis (*n*; %)	87; 42%	16; 18%	71; 82%	0.7095
Pleuropulmonary sepsis (*n*; %)	29; 14%	6; 21%	23; 79%	0.5960
Urinary tract infections (*n*; %)	35; 17%	3; 9%	32; 91%	0.2157
Skin and soft tissue infections (*n*; %)	35; 17%	8; 23%	27; 77%	0.3280
Other sepsis (*n*; %)	19; 10%	2; 11%	17; 89%	0.5397
KDIGO AKI stage max, med (IQR)	1 (0–3)	3 (2–3)	1 (0–2)	< 0.0001
SAPSII score day 1, med (IQR)	57 (40–68)	75 (64–89)	54 (38–63)	< 0.0001
SOFA day 1, med (IQR)	7 (4–9)	10 (7–12)	6 (4–8)	< 0.0001
Lactate day 1 (mmol/l), med (IQR)	2.2 (1.4–3.4)	3.0 (2.0–5.3)	2.1 (1.3–3.3)	0.0002
Norepinephrine day 1 (micg/kg/min), med (IQR)	0.23 (0.0–0.55)	0.66 (0.48–1.24)	0.18 (0–0.41)	< 0.0001
RRT day 1 (*n*; %)	22; 11%	9; 26%	13; 8%	0.0044
SOFA day 3, med (IQR)	4 (1–7)	11 (7–13)	3 (1–6)	< 0.0001
Lactate day 3 (mmol/l), med (IQR)	1.4 (1.0–1.9)	2.4 (1.8–6.9)	1.3 (0.9–1.8)	< 0.0001
Norepinephrine day 3 (micg/kg/min), med (IQR)	0.0 (0.0–0.24)	0.48 (0.25–0.91)	0.0 (0.0- 0.1)	< 0.0001
RRT day 3 (*n*; %)	25; 12%	12; 34%	13; 8%	0.0001
Length of stay in ICU (days), med (IQR)	7 (3–16)	6 (3–13)	7 (4–17)	0.0724
Length of mechanical ventilation (days), med (IQR)	2 (0–8)	5 (2–12)	2 (0–7)	0.0007
Days alive without MV at D28, med (IQR)	26 (11–28)	0 (0–2)	26 (21–28)	< 0.0001

Stratification according to mortality at day 28. Mann–Whitney's test was used for continuous variables, and Fisher's exact test was used for categorical variables

*KDIGO* Kidney Disease: Improving Global Outcomes, *SAPSII* Simplified Acute Physiology Score II, *SOFA* Sepsis-related Organ Failure Assessment, *RRT* renal replacement therapy, *MV* mechanical ventilation

Moreover, concerning feeding (oral, enteral, parenteral, or both enteral/parenteral nutrition), 48% of the patients received nutrition at admission, 88% at day 3, and 91% at ICU discharge.

### b. Lipoprotein concentration over time

Of the 205 patients included, 101 had a previous lipid test performed in the absence of any infectious symptoms (= prior). The median interval between this previous assessment and admission of the patient to the ICU was 10 (5–24) months. Except for habitual statin use, there were no differences in general characteristics or outcomes between patients with and without previous lipid tests (see Additional file 1: S1).

**Fig. 1:**
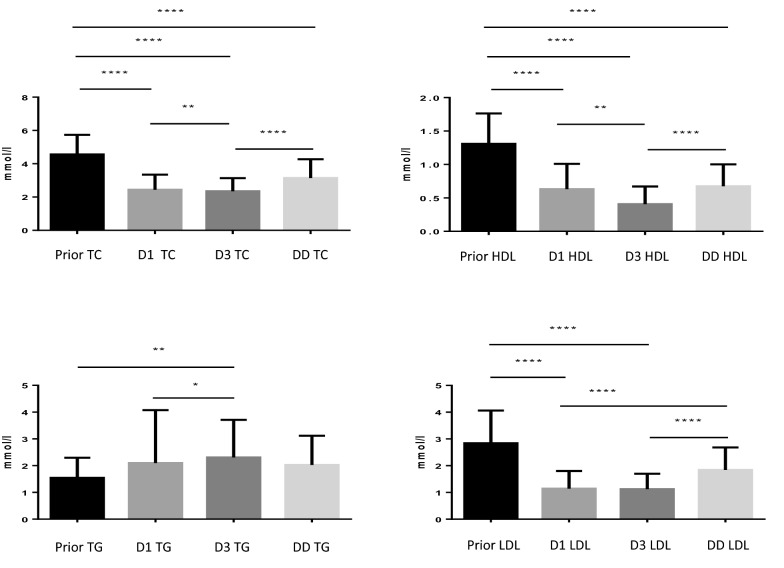
Variation in lipoprotein levels as a function of time (Friedman's test). *: *p* < 0.05; **: *p* < 0.01; ***: *p* < 0.001; ****: *p* < 0.0001. *TC* total cholesterol, *HDL* high-density lipoprotein, *LDL* low-density lipoprotein, *TG* triglycerides, *D1* Day 1, *D3* Day 3, *DD* discharge day

Figure 1 shows changes in lipoprotein concentrations with time, taking into account values prior to ICU hospitalization. To compare the different sampling times (prior, day 1, day 3, and discharge day), Friedman's test with repeated measurements was necessary. Repeated measurements required us to have the data at each time (prior, D1, D3, and DD). In this context, we included 66 patients in the analysis. This analysis showed a decrease in HDL-C concentration between the prehospitalization value and day 1 followed by a decrease at day 3. The concentration on the day of discharge was higher than that on day 3, but it did not reach the level of the prehospitalization assessment. Similar variations were found for total cholesterol and LDL-C values but not for triglycerides.

### c. Outcome

Table 1 stratifies general patient characteristics according to mortality at day 28. All deaths at 28 days were caused by multiorgan failure related to sepsis. Patients who died at day 28 were older [67 (62–77) years vs. 63 (51–72) years, *p* = 0.0208] and had higher Kidney Disease: Improving Global Outcomes scores [3 (2–3) vs. 1 (0–2), *p* < 0.0001] and Simplified Acute Physiology Score II and Sepsis-related Organ Failure Assessment scores at admission [75 (64–89) vs. 54 (38–63), *p* < 0.0001 and 10 (7–12) vs. 6 (4–8), *p* < 0.0001, respectively], higher plasma lactate levels at admission [3.0 (2.0–5.3) vs. 2.1 (1.3–3.3) mmol/l, *p* = 0.0002], higher doses of norepinephrine at admission [0.66 (0.48–1.24) µg/kg/min vs. 0.18 (0.0–0.41), *p* < 0.0001], and greater use of renal replacement therapy (26% vs. 8%, *p* = 0.0044). The length of mechanical ventilation was also greater in the group of patients who died at 28 days [5 (2–12) vs. 2 (0–7) days, *p* = 0.0007]. Similarly, patients who died had fewer alive days without mechanical ventilation between admission and day 28 than survivors at day 28 [0 (0–2) days vs. 26 (11–28) days, *p* < 0.0001].

### d. Relationship between lipoprotein concentrations and patient outcomes

**Fig. 2:**
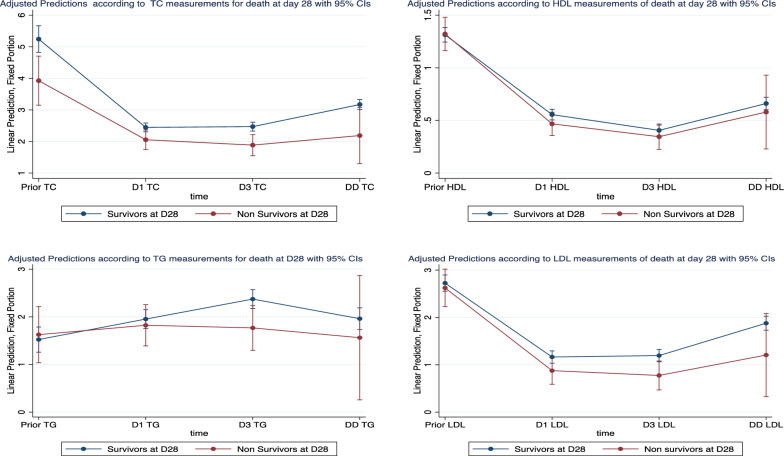
Margin plot of the mixed model for repeated measures adjusted predictions of lipoprotein levels for mortality at day 28 taking into account prior lipid values. Except in the case of triglycerides, all models were statistically significant (*p* < 0.0001). *TC* total cholesterol, *HDL* high-density lipoprotein, *LDL* low-density lipoprotein, *TG* triglycerides, *D1* Day 1, *D3* Day 3, *DD* discharge day

Mixed models for repeated measures of lipoprotein concentrations as a predictor of mortality showed a significant difference in HDL-C and LDL-C concentrations over time (prior sample, day 1, day 3, and discharge day) between survivors and nonsurvivors (Fig. 2). Another mixed model for repeated measures of lipoprotein concentrations as predictors of mortality focusing on day 1, day 3, and discharge day also underlined a significant difference in HDL-C and LDL-C concentrations over time between survivors and nonsurvivors (Additional file 2: S2).

**Fig. 3:**
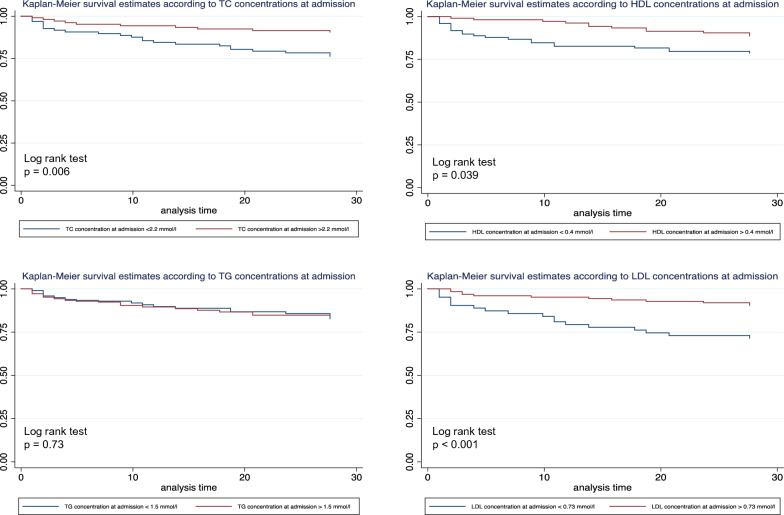
Kaplan–Meier estimates of survival in the 28 days after the onset of sepsis for patients with different initial levels of lipoproteins. *TC* total cholesterol, *HDL* high-density lipoprotein, *LDL* low-density lipoprotein, *TG* triglycerides. *N* = 205 patients were included in these analyses

Figure 3 shows mortality at day 28 as a function of total cholesterol, HDL-C, LDL-C, and triglyceride concentrations. Mortality at day 28 of patients with total cholesterol concentrations less than 2.2 mmol/l at admission was significantly higher (log-rank test, *p* = 0.006). Mortality at day 28 of patients with HDL-C concentration levels below 0.4 mmol/l at admission was significantly higher (log-rank test, *p* = 0.034). Mortality at day 28 of patients with LDL-C concentrations less than 0.73 mmol/l at admission was significantly higher (log-rank test, *p* < 0.001). No difference in mortality was found with respect to triglyceride concentrations at admission.

**Fig. 4:**
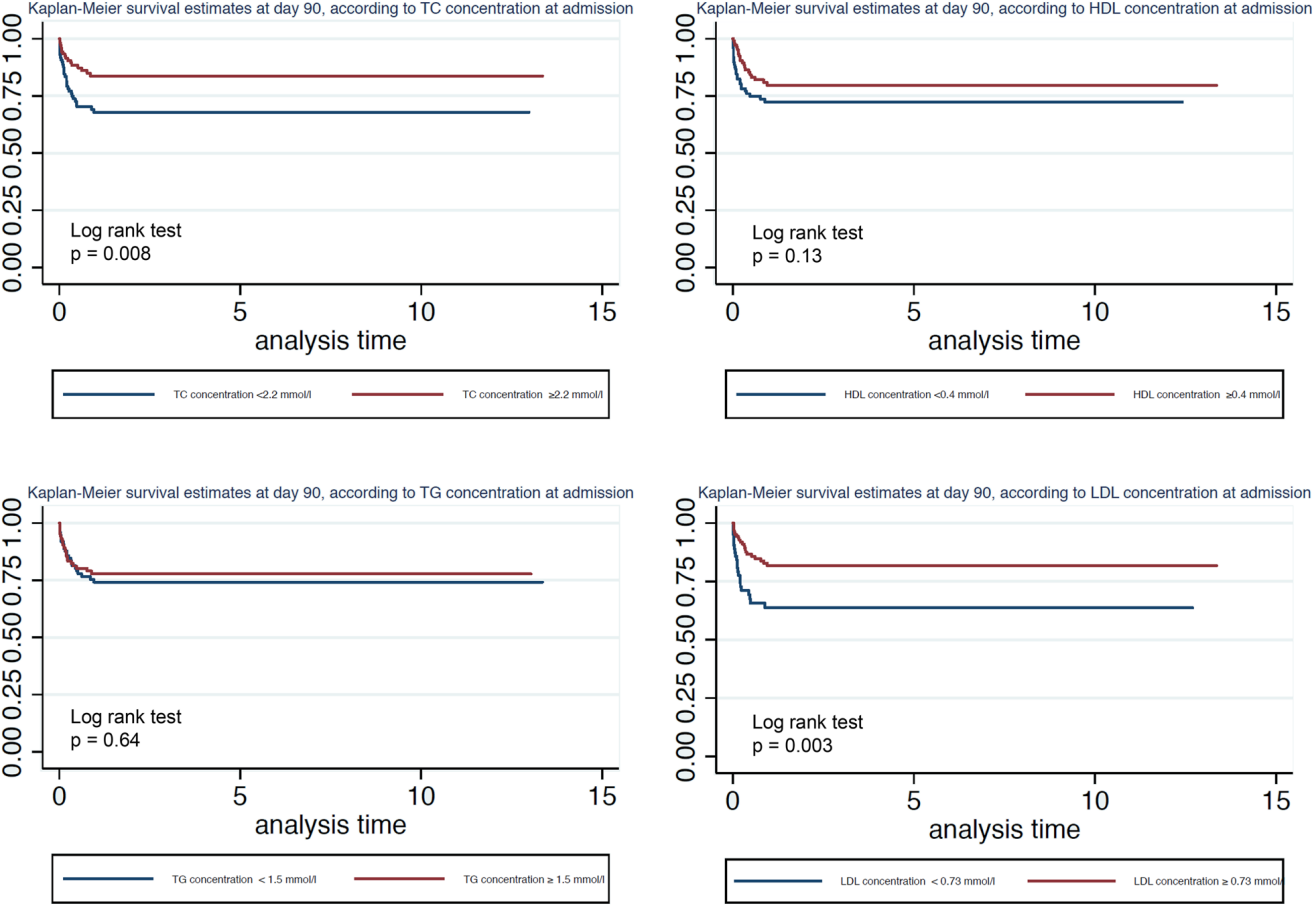
Kaplan–Meier estimates of survival in the 90 days after the onset of sepsis for patients with different initial levels of lipoproteins. *TC* total cholesterol, *HDL* high-density lipoprotein, *LDL* low-density lipoprotein, *TG* triglycerides. *N* = 205 patients were included in these analyses

Figure 4 shows mortality at day 90 as a function of the total cholesterol, HDL-C, LDL-C, and triglyceride concentrations. Mortality at day 90 of patients with total cholesterol concentrations less than 2.2 mmol/l at admission was significantly higher (log-rank test, *p* = 0.008). Mortality at day 90 of patients with LDL-C concentrations less than 0.73 mmol/l at admission was significantly higher (log-rank test, *p* = 0.003). Interestingly, no difference in mortality at day 90 was found with respect to HDL-C concentration at admission (log-rank test, *p* = 0.13). No difference in mortality was found with respect to triglyceride concentrations at admission.

**Fig. 5:**
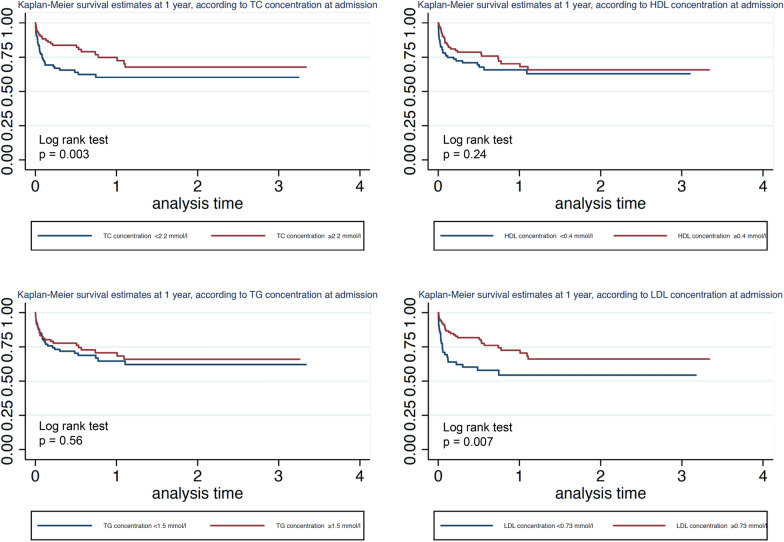
Kaplan–Meier estimates of survival 1 year after the onset of sepsis for patients with different initial levels of lipoproteins. *TC* total cholesterol, *HDL* high-density lipoprotein, *LDL* low-density lipoprotein, *TG* triglycerides. *N* = 205 patients were included in these analyses

Figure 5 shows mortality at 1 year as a function of total cholesterol, HDL-C, LDL-C, and triglyceride concentrations. Mortality at 1 year of patients with total cholesterol concentrations less than 2.2 mmol/l at admission was significantly higher (log-rank test, *p* = 0.003). Mortality at 1 year of patients with LDL-C concentrations less than 0.73 mmol/l at admission was significantly higher (log-rank test, *p* = 0.007). Interestingly, no difference in mortality at 1 year was found with respect to HLD-C concentration at admission (log-rank test, *p* = 0.24). No difference in mortality was found with respect to triglyceride concentration at admission.

### e. Relationship between lipoprotein concentrations and outcome in patient subgroups

Additional file 3: S3 shows mortality at day 28 as a function of total cholesterol, HDL-C, LDL-C, and triglyceride concentrations in the specific subgroup of septic shock patients. Mortality at day 28 of septic shock patients with total cholesterol concentrations less than 2.2 mmol/l at admission was significantly higher (log-rank test, *p* < 0.001). Mortality at day 28 of septic shock patients with HDL-C concentrations less than 0.4 mmol/l at admission was significantly higher (log-rank test, *p* < 0.001). Mortality at day 28 of patients with LDL-C concentrations less than 0.73 mmol/l at admission was significantly higher (log-rank test, *p* = 0.002). No difference in mortality related to triglyceride concentrations at admission was found in this subgroup.

According to the log-rank test, low levels of LDL-C and HDL-C at admission were statistically associated with 28-day mortality in the septic pleuropneumonia and bacteremia subgroups. In the intra-abdominal sepsis subgroup, LDL-C concentration at admission < 0.73 mmol/l was associated with higher mortality. No difference in mortality related to HDL-C levels was found in intra-abdominal sepsis patients (Additional file 4: S4).

### f. Correlation between prior lipoprotein levels and patient outcomes

**Fig. 6:**
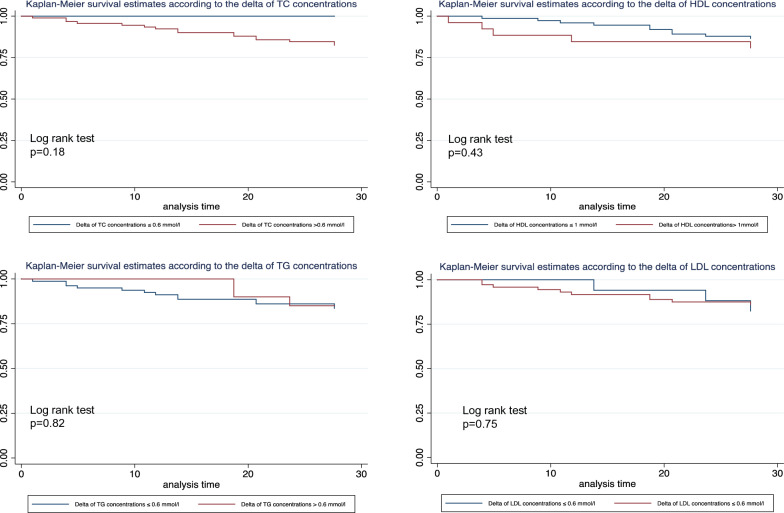
Kaplan–Meier estimates of survival in the 28 days after the onset of sepsis according to the differences between patients’ prior concentrations of lipoproteins and those at day one. *TC* total cholesterol, *HDL* high-density lipoprotein, *LDL* low-density lipoprotein, *TG* triglycerides. *N* = 101 patients were included in these analyses

The differences (delta) in lipoprotein values between prehospitalization and day-1 measurements were compared between survivors and nonsurvivors at day 28. According to the Kaplan–Meier curves, there was no significant association between any of these differences and mortality at day 28 (delta total cholesterol, log-rank test *p* = 0.18; delta HDL-C, log-rank test *p* = 0.43; delta LDL-C, log-rank test *p* = 0.75; and delta triglycerides, log-rank test *p* = 0.82). The results are expressed in Fig. 6.

## Discussion

The analysis of this cohort of 205 patients hospitalized in an ICU for septic shock or severe sepsis found the following:

- a decrease in HDL-C and LDL-C concentrations compared to prior levels and a significant difference over time during ICU hospitalization between survivors and nonsurvivors;
- a statistically significant association between HDL-C concentration and mortality at day 28 but not at day 90 or at 1 year;
- a statistically significant association between LDL-C concentration and short- and long-term mortality (at day 28, day 90, and at 1 year); and
- no link between lipoprotein levels measured prior to hospitalization and the outcome of the patients.

Here, we did not find a relationship between lipoprotein levels prior to hospitalization and the patient outcome. Only modifications of these particles under septic conditions seem to be linked to the patients’ outcome. Our findings are not in accordance with two recent studies that underlined a potential link between lipoprotein basal level and sepsis outcome. A retrospective single-center cohort study involving 3,592 septic patients based on electronic medical records showed that baseline values for both LDL-C and triglycerides were associated with mortality when using a SIRS-based definition of sepsis [38]. Trinder et al. analyzed the effect of HDL-C, LDL-C, and triglyceride polygenic scores on survival in 3222 participants in a UK biobank who were hospitalized for sepsis [39]. Interestingly, the authors found that there was a significant inverse association between continuous HDL-C polygenic score and 28-day mortality, whereas LDL-C and triglyceride polygenic scores were not associated with sepsis mortality. Even if these two retrospective studies based on electronic medical records found controversial results, they highlighted that basal lipoprotein levels or genetic-based analyses could have a link with the outcome in the case of sepsis. The differences found in our study compared with Maile et al.’s and Trinder et al.’s studies could be explained by our population consisting strictly of ICU patients. Additionally, our study took into account basal crude lipoprotein concentrations and not genetic-based analyses.

The low concentration of HDL-C at ICU admission found in our cohort is consistent with the results of other studies conducted in septic patients [23–28, 30, 31]. The mechanism that leads to the decreased concentration of HDL-C during the septic state is poorly described [40]. Several hypotheses have been proposed to explain this decrease: there is a significant consumption of HDL particles, a consequent hemodilution, a decrease in HDL synthesis by the liver, particularly in the case of associated liver dysfunction, or an increase in HDL clearance following an increase in scavenger receptor class B type 1 (SRB-1) expression [41]. The increase in HDL-C levels over time and the healing of the infectious process that we observed with improvement in the patient's clinical condition are possibly explained by bacterial clearance, restoration of normovolemia, and a drastic decrease in capillary leakage associated with improvement of the microcirculatory network; they could also be explained by an increase in hepatic HDL synthesis and appropriate regulation of SRB-1 expression [11, 40].

The link between lipoprotein concentrations and patient outcome is controversial, according to previous studies. We found in our cohort of septic ICU patients that low HDL-C and LDL-C concentrations at admission are strongly associated with increased mortality at day 28, in accordance with several other studies in the field [24–27]. Interestingly, the long-term mortality relationship is more controversial. Although there was a statistically significant association between LDL-C concentration at 90 days and at 1 year, no link was found between HDL-C concentration and long-term mortality. To our knowledge, only one study on lipoproteins during sepsis has focused on long-term outcomes [42]. Roveran Genga et al. showed that a composite end-point of death or progression to a decreased estimated glomerular filtration rate at 2 years was higher in patients with HDL-C concentrations less than 33.06 mg/dl during a septic episode.

This link with the long-term outcome is not very well characterized. These findings could be explained by some extrinsic factors that could independently influence mortality, such as basal medical conditions or other morbidity issues interfering with the outcome but without any link with the initial septic episode. Further studies focusing on this long-term mortality seem to be of interest.

Although HDL-C concentration is a parameter that appears to be associated with short-term patient outcome, knowledge of the functionality of these lipoproteins under septic conditions could potentially help us to better characterize our patients. Trinder et al. identified a rare missense variant in cholesteryl ester transfer protein (CETP) that was associated with a significant reduction in HDL-C levels during sepsis [43]. Recently, in a population of septic patients, we described a shift toward large HDL particles compared to nonseptic patients, an observation that may reflect a potential dysfunction of these particles [44]. Other studies emphasized that changes in HDL-C efflux capacity or HDL oxidation are potentially correlated with poor outcome [45, 46]. Based on these findings, we think that lipoprotein concentrations are potentially not sufficient to characterize patient outcome. Studies designed to better evaluate these dysfunctions, such as lipidomic or proteomic analyses, should be conducted in the future.

In this context, therapy aiming to restore lipoprotein levels during septic shock episodes might be interesting to explore. Thus, infusion of functional particles could be interesting to test. Some experimental studies evaluated the efficacy of reconstituted HDL injection and apolipoprotein A-I mimetic peptide injection in animal models of sepsis [16–20]. These studies have shown a positive effect of these particles, resulting in decreased animal morbidity and mortality and decreased levels of systemic and histological inflammatory markers. All of these positive experimental findings are very encouraging and may lead to testing of these particles in human trials [40].

CETP inhibitors increase HDL-C levels by decreasing the transfer of cholesteryl esters to other lipoprotein particles, such as LDL. Although the results of recent phase 3 randomized controlled trials testing CETP inhibitors (torcetrapib, dalcetrapib, evacetrapib and anacetrapib) in atherosclerotic patients are very disappointing, such therapeutics might be interesting to evaluate because of the potential increase in the level of HDL-C [47–49].

No adjuvant therapy aimed at restoring LDL-C levels has been tested. However, because LDL particles can neutralize lipopolysaccharides and increase bacterial clearance, the LDL receptor could also be an interesting target. In this context, proprotein convertase subtilisin/kexin type 9 (PCSK9) inhibitors enhance LDL-mediated elimination of lipopolysaccharide and have shown promise in preclinical studies of sepsis [50, 51]. A phase 2 study testing a PCSK9 inhibitor, evolocumab, in septic patients is currently underway [52].

Our study has several limitations:

- First, it is a monocentric study conducted in a surgical ICU with a majority of patients with abdominal sepsis; in this respect, it does not reflect the conventional recruitment of medico-surgical ICUs.
- Second, in our study, we did not measure biomarkers of inflammation, such as cytokines, and therefore were unable to compare patients according to their inflammatory state.
- Third, since patients with a previous lipid profile are more likely to be treated with statins, this may lead to a number of biases. Nevertheless, mixed models for repeated measures adjusted predictions of lipoprotein levels of mortality at day 28 taking into account or not the prior basal lipoprotein concentrations gave the same results (i.e., significant differences in HDL-C and LDL-C concentrations over time between survivors and nonsurvivors).
- Fourth, because of the nature of this prospective cohort study, we did not calculate the number of patients to include. The number of patients with a prior lipid analysis was difficult to predict. We consider that this part of the results is underpowered, and conclusions on the lipid concentrations in prior sepsis remain to be confirmed in additional studies addressing this issue.
- Fifth, the contradictory results concerning the link between lipoprotein concentration during the sepsis state and outcome (short- and long-term) merit further attention to identify these long-term outcome parameters.

## Conclusion

Our study of 205 septic patients emphasizes the strong link between low HDL-C and LDL-C concentrations in ICU patients and 28-day mortality, even if the relationship with long-term outcome remains controversial. We found that patients’ HDL-C and LDL-C concentrations prior to hospitalization did not show correlations with the outcome of these patients. These results confirm the positive functions of these lacking lipoproteins in the septic state. The relationship between lipoproteins and long-term outcome parameters nonetheless merits further attention.

The observed depletion of lipoproteins, especially that of HDL particles, at the early phase of sepsis emphasizes the need to evaluate the key role of these particles. Further experimental studies investigating the functionalities of these particles under septic conditions are thus crucial.

## Supplementary Information

**Additional file 1: S1.** Comparison between patients with or without prior lipid profiles. The results are expressed as percentages for categorical variables and medians (interquartiles) for continuous variables [95% CI]. D1: day 1; SOFA score: Sequential Organ Failure Assessment score; SAPSII score: Simplified acute physiology II score; KDIGO AKI stage: Kidney Disease: Improving Global Outcomes Acute Kidney Injury stage; RRT: renal replacement therapy; ICU: Intensive Care Unit.

**Additional file 2: S2.** Margins plot of the mixed model for repeated measures adjusted predictions of lipoprotein levels for mortality at day 28. All models were statistically significant (p < 0.0001). TC: Total cholesterol; HDL: High-density lipoprotein; LDL: Low-density lipoprotein; TG: Triglycerides; D1: Day 1; D3: Day 3, DD: Discharge day.

**Additional file 3: S3.** Kaplan–Meier estimates of survival in the 28 days after the onset of sepsis for patients with different initial levels of lipoproteins in the septic shock subgroup. TC: Total cholesterol; HDL: High-density lipoprotein; LDL: Low-density lipoprotein; TG: Triglycerides.

**Additional file 4: S4.** Kaplan–Meier estimates of survival in the 28 days after the onset of sepsis for patients with different initial levels of high-density lipoprotein cholesterol and low-density lipoprotein cholesterol in the bacteremia, intra-abdominal sepsis, and pleuropulmonary sepsis subgroups. HDL: High-density lipoprotein; LDL: Low-density lipoprotein.

## Data Availability

The datasets used and analyzed in this study are available from the corresponding author upon reasonable request.

## Abbreviations

CETP: Cholesteryl ester transfer protein
HDL: High-density lipoprotein
ICU: Intensive care unit
KDIGO: Kidney Disease: Improving Global Outcomes
LDL: Low-density lipoprotein
LPS: Lipopolysaccharides
LTA: Lipoteichoic acid
MV: Mechanical ventilation
PCSK9: Proprotein convertase subtilisin/kexin type 9
RRT: Renal replacement therapy
SAPSII: Simplified Acute Physiology Score II
SOFA: Sepsis-related Organ Failure Assessment
SRB-1: Scavenger receptor class B type 1
TC: Total cholesterol
TG: Triglyceride
