# Supplementary material for: Relationship between lipoprotein concentrations and short-term and 1-year mortality in intensive care unit septic patients: results from the HIGHSEPS study
Source: Ann Intensive Care. 2021 Jan 19;11:11. doi: 10.1186/s13613-021-00800-0 (PMC7815878; doi:10.1186/s13613-021-00800-0)
Supplement: Supplementary file 1 — Additional file 1: S1. Comparison between patients with or without prior lipid profiles. The results are expressed as percentages for categorical variables and medians (interquartiles) for continuous variables [95% CI]. D1: day 1; SOFA score: Sequential Organ Failure Assessment score; SAPSII score: Simplified acute physiology II score; KDIGO AKI stage: Kidney Disease: Improving Global Outcomes Acute Kidney Injury stage; RRT: renal replacement therapy; ICU: Intensive Care Unit. [file 13613_2021_800_MOESM1_ESM.docx]

|  | **Patients without anterior lipid profile (n=104)** | **Patients with anterior lipid profile (n=101)** | **Odds ratio** | **p** |
| --- | --- | --- | --- | --- |
| **Male (%)** | 51 | 54 | 1.14 [0.66;1.98] | 0.67 |
| **Age (years)** | 60 (48-74) | 64 (54-72) | 1.00 [0.99;1.02] | 0.37 |
| **Weight (kg)** | 75 (60-87) | 81 (67-90) | 1.00 [0.99;1.02] | 0.054 |
| **Statins (%)** | 20 | 42 | 2.90 [1.55;5.43] | 0.001 |
| **SOFA D1** | 7 (4-10) | 7(4-9) | 1.01 [0.94;1.09] | 0.69 |
| **SAPSII D1** | 54 (40-67) | 56 (41-69) | 1.00 [0.99;1.02] | 0.47 |
| **KDIGO max** | 1 (0-3) | 2(1-3) | 1.10 [0.87;1.39] | 0.42 |
| **Lactate D1 (mmol/l)** | 3.0 (1.4-3.3) | 3.0 (1.3-3.7) | 0.99 [0.90;1.11] | 0.99 |
| **Septic shock (%)** | 70 | 75 | 1.29 [0.70;2.39] | 0.41 |
| **Severe sepsis (%)** | 30 | 25 | 0.73 [0.39;1.36] | 0.32 |
| **RRT at D1 (%)** | 11.8 | 9.7 | 0.80 [0.33;1.95] | 0.63 |
| **Norepinephrine at D1 (%)** | 69 | 70 | 1.06 [0.58;1.92] | 0.88 |
| **Bacteremia (%)** | 28 | 23 | 0.76 [0.41;1.43] | 0.40 |
| **Length of mechanical ventilation (day)** | 2 50-8) | 2 (0-8) | 1.01 [0.98;1.04] | 0.27 |
| **Length of stay in ICU (day)** | 6 (3-14) | 8 (5-19) | 1.01 [0.99;1.03] | 0.10 |
| **Mortality at day28 (%)** | 17.6 | 16.5 | 0.92 [0.44;1.91] | 0.85 |
