# Supplementary figures and images for: Relationship between lipoprotein concentrations and short-term and 1-year mortality in intensive care unit septic patients: results from the HIGHSEPS study
Source: Ann Intensive Care. 2021 Jan 19;11:11. doi: 10.1186/s13613-021-00800-0 (PMC7815878; doi:10.1186/s13613-021-00800-0)

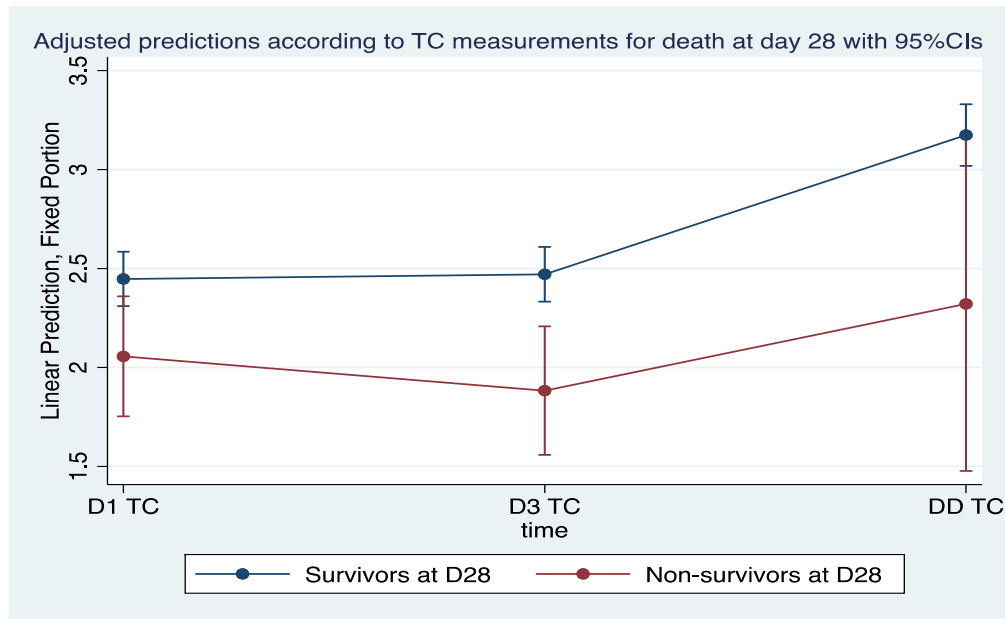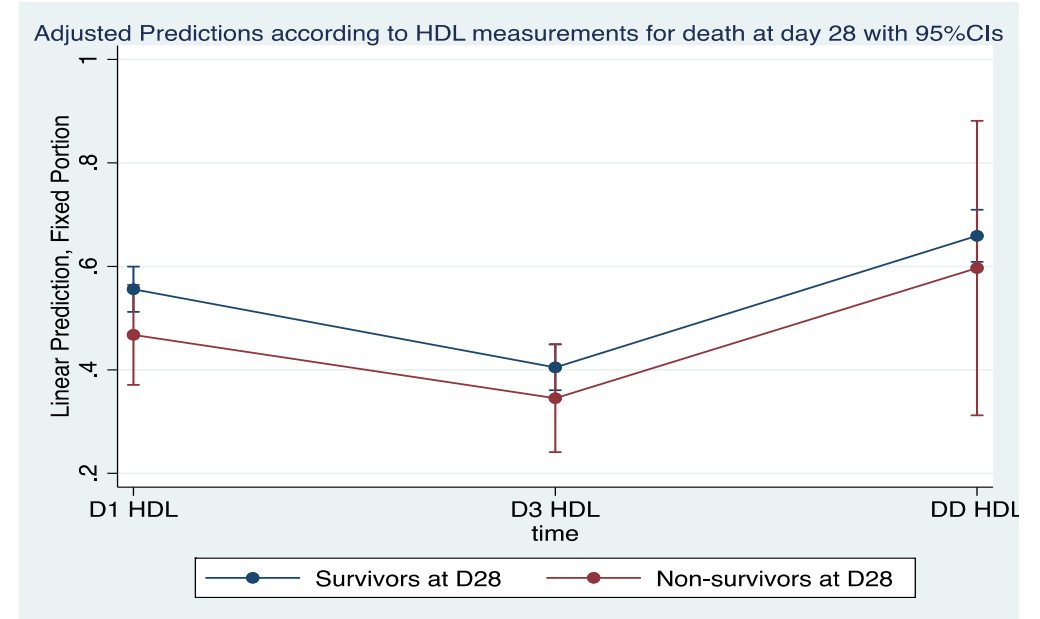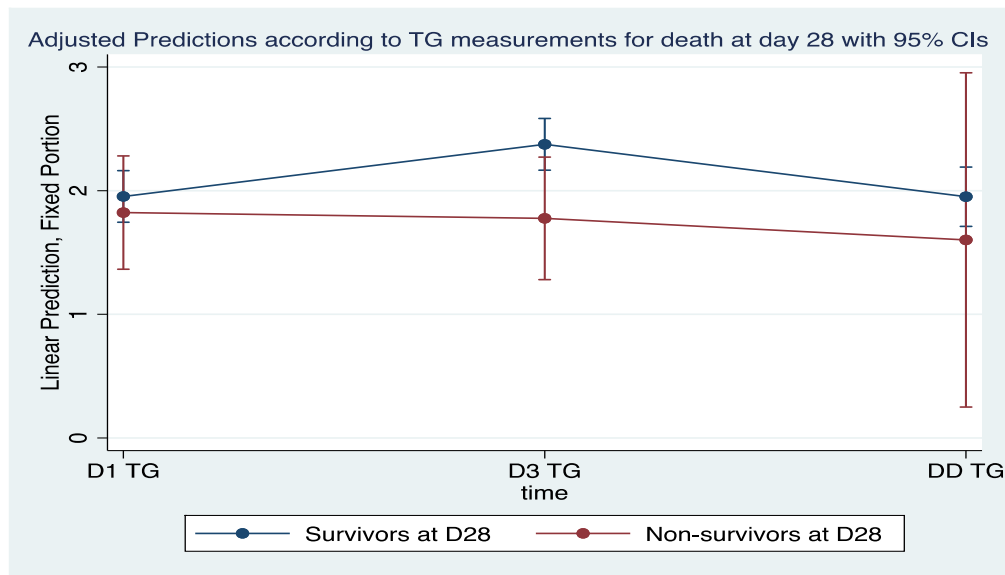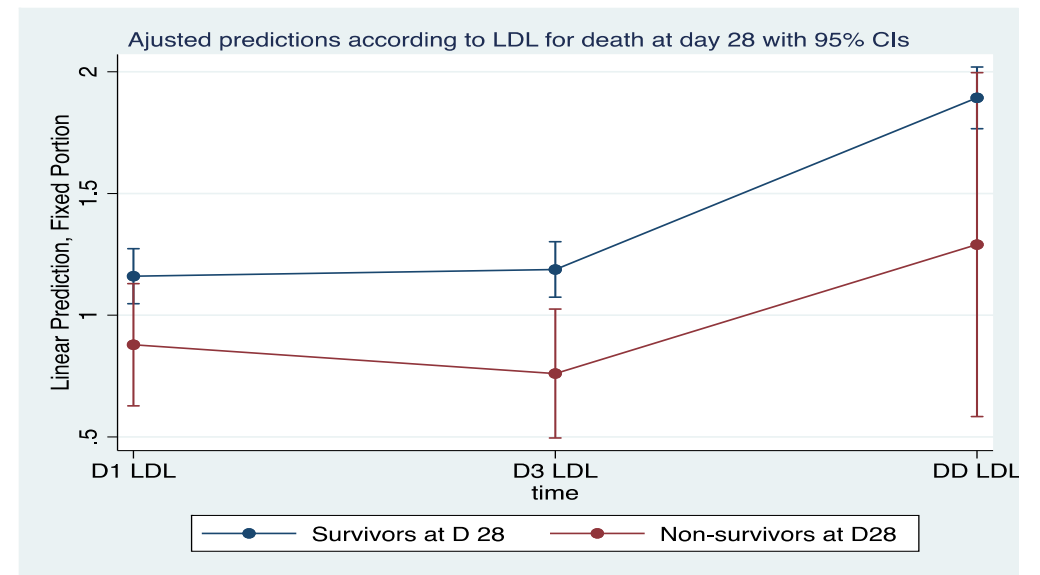

Supplement: Supplementary file 2 — Additional file 2: S2. Margins plot of the mixed model for repeated measures adjusted predictions of lipoprotein levels for mortality at day 28. All models were statistically significant (p < 0.0001). TC: Total cholesterol; HDL: High-density lipoprotein; LDL: Low-density lipoprotein; TG: Triglycerides; D1: Day 1; D3: Day 3, DD: Discharge day. [file 13613_2021_800_MOESM2_ESM.pdf]

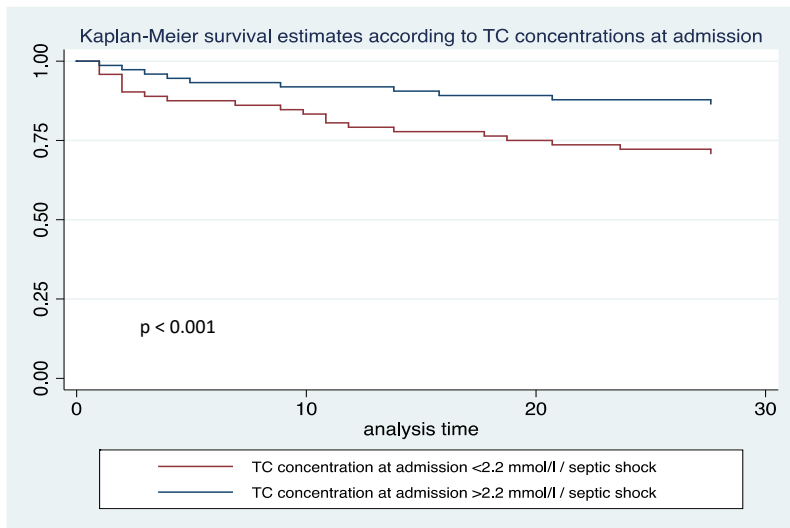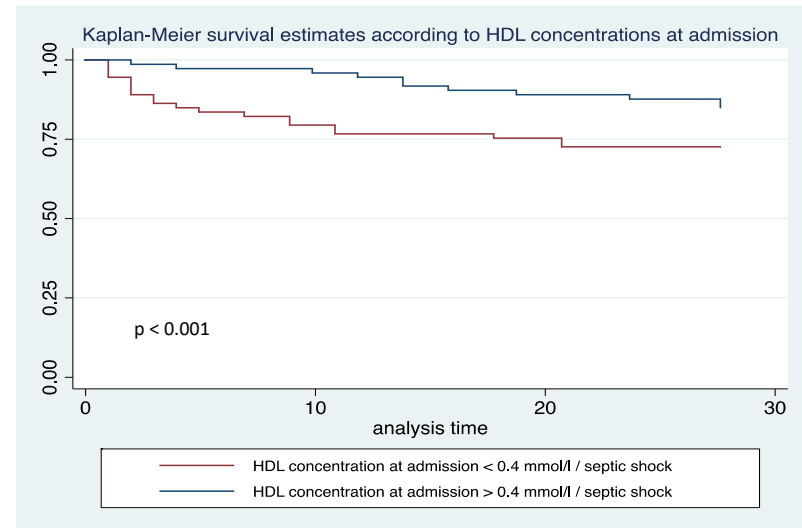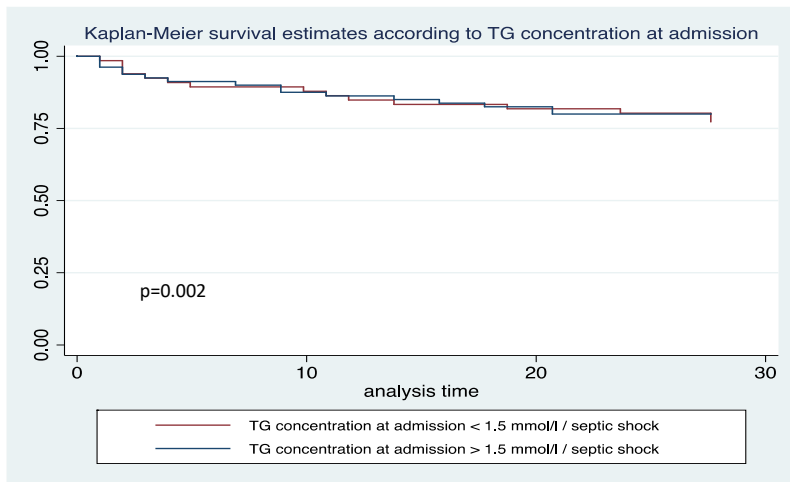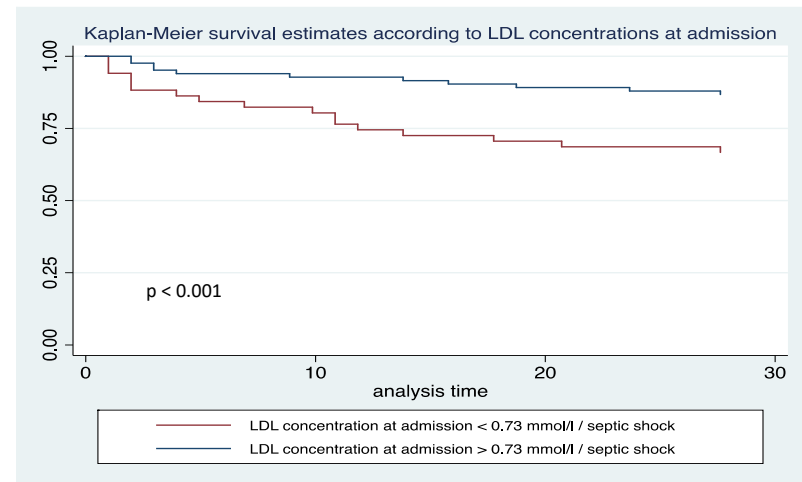

Supplement: Supplementary file 3 — Additional file 3: S3. Kaplan–Meier estimates of survival in the 28 days after the onset of sepsis for patients with different initial levels of lipoproteins in the septic shock subgroup. TC: Total cholesterol; HDL: High-density lipoprotein; LDL: Low-density lipoprotein; TG: Triglycerides. [file 13613_2021_800_MOESM3_ESM.pdf]

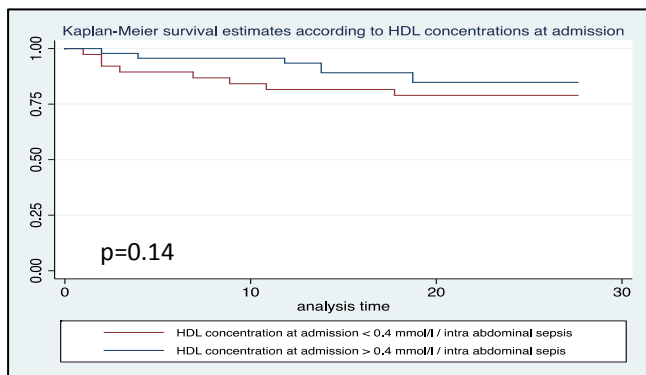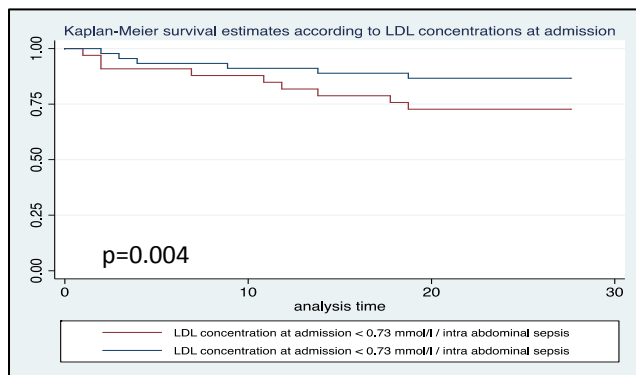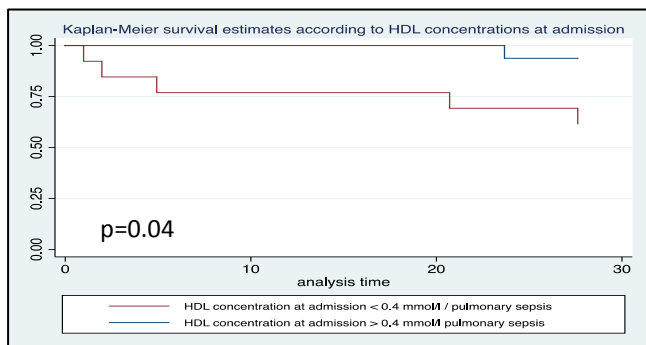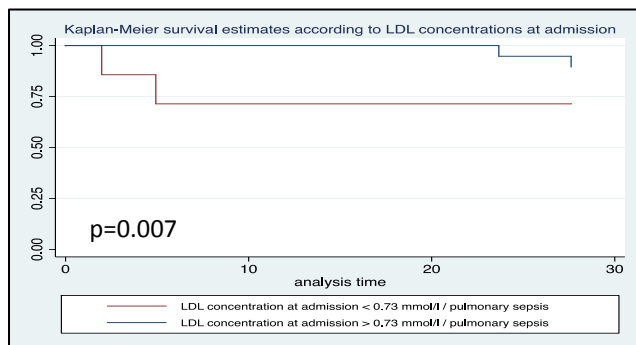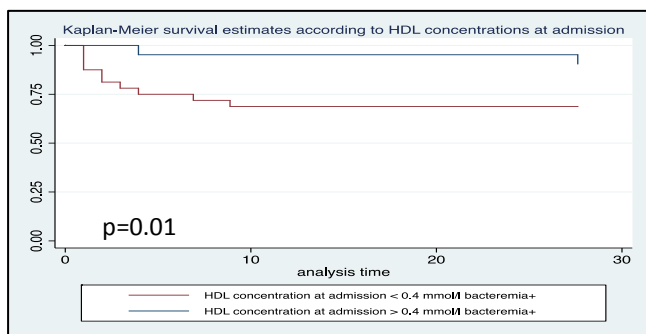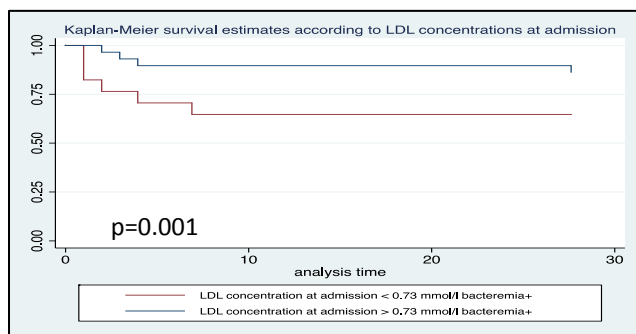

Supplement: Supplementary file 4 — Additional file 4: S4. Kaplan–Meier estimates of survival in the 28 days after the onset of sepsis for patients with different initial levels of high-density lipoprotein cholesterol and low-density lipoprotein cholesterol in the bacteremia, intra-abdominal sepsis, and pleuropulmonary sepsis subgroups. HDL: High-density lipoprotein; LDL: Low-density lipoprotein. [file 13613_2021_800_MOESM4_ESM.pdf]
